# Non-Classical Spring-to-Summer Phytoplankton Community Reorganization in Liaodong Bay Associated with Shifts in Phosphorus Regime and Nutrient Stoichiometry

**DOI:** 10.3390/microorganisms14081611

**Published:** 2026-07-23

**Authors:** Baozhan Liu, Zhaohui Wang, Minghao Yin, Yanqing Li, Xiansheng Zhang, Guangjun Song, Xin Song, Ruiqiang Shi, Yan Liu, Jinhao Wu

**Affiliations:** 1 Center of Eco-Environmental Monitoring and Scientific Research, Administration of Ecology and Environment of Haihe River Basin and Beihai Sea Area, Ministry of Ecology and Environment of People’s Republic of China, Tianjin 300170, China; 2 Liaoning Ocean and Fisheries Science Research Institute, Dalian 116023, China; 3 Dalian Ocean Development Affairs Service Center, Dalian 116000, China

**Keywords:** phytoplankton community, *Noctiluca scintillans*, phosphorus forms, nutrient stoichiometry, seasonal reorganization, Liaodong Bay

## Abstract

In temperate coastal waters, phytoplankton communities often follow a classical seasonal pattern, with diatoms dominating in spring and dinoflagellates becoming more prominent during summer. However, spring-to-summer phytoplankton community reorganization in eutrophic Liaodong Bay remains poorly characterized. Here, we examined seasonal changes in phytoplankton communities and environmental conditions in Liaodong Bay in spring and summer 2025. The phytoplankton community underwent a marked spring-to-summer reorganization that differed from the classical seasonal pattern. In spring, dinoflagellates accounted for 63.51% of total community abundance, with the heterotrophic dinoflagellate *N. scintillans* comprising 99.56% of this fraction. By contrast, diatoms comprised 91.02% of the summer community, with *Coscinodiscus granii*, *Skeletonema costatum*, and other diatom taxa sharing dominance. This reorganization coincided with a seasonal shift in phosphorus regime and nutrient stoichiometry, from low dissolved inorganic phosphorus (DIP; 0.12 μmol P L⁻¹), dominance of dissolved organic phosphorus (DOP; 0.28 μmol P L⁻¹) and high N:P in spring to higher DIP (0.66 μmol P L^−1^), lower DOP (0.18 μmol P L^−1^), lower N:P, and higher Si:N in summer. Correlation and redundancy analyses indicated that the spring *N. scintillans*-dominated assemblage was broadly aligned with DOP-rich and high-N:P conditions, whereas summer-dominant diatoms were broadly aligned with DIP, dissolved silicate (DSi), and Si:N gradients, although not all bivariate relationships were significant. These patterns suggest that potential relative inorganic phosphorus deficiency in spring may have been less favorable for diatom dominance, whereas higher DIP, lower N:P, and higher Si:N in summer were consistent with a resource regime more favorable for multi-diatom expansion. Overall, the phytoplankton community in Liaodong Bay exhibited a non-classical seasonal reorganization from a spring *N. scintillans*-dominated assemblage, rather than a conventional autotrophic dinoflagellate-dominated stage, to summer multi-diatom co-dominance, coinciding with shifts in phosphorus regime and nutrient stoichiometry.

## 1. Introduction

Phytoplankton are major contributors to primary production in marine ecosystems, particularly in estuarine and coastal waters, where community composition and seasonal dynamics regulate carbon fixation, biogeochemical cycling, and food-web structure [1,2]. In temperate coastal waters, phytoplankton communities often show a recurrent seasonal pattern, with diatoms dominating in spring and dinoflagellates becoming more prominent in summer. This transition is generally associated with rising temperature, enhanced water-column stratification, and progressive depletion of surface inorganic nutrients [3]. For example, spring blooms in the Inner Oslofjord are typically dominated by diatoms, followed by a gradual increase in dinoflagellates and other phytoplankton groups [3]. Similarly, long-term observations from the central Bohai Sea indicate that phytoplankton communities are generally diatom-dominated in spring under cooler conditions, whereas dinoflagellates become more prominent with summer warming [4]. However, in eutrophic coastal waters strongly influenced by human activities, particularly estuaries and semi-enclosed bays, seasonal phytoplankton transitions do not always follow this common trajectory. For instance, atypical seasonal succession patterns have been reported in coastal waters off Nan’ao Island and in the Baltic Sea, where dinoflagellates may dominate during periods that are typically dominated by diatoms, rather than following the classical spring diatom–summer dinoflagellate succession pattern [5,6]. These observations suggest that seasonal phytoplankton community organization in coastal environments is highly context-dependent and may deviate from canonical patterns in eutrophic semi-enclosed systems.

Although the mechanisms underlying such non-classical seasonal patterns remain incompletely understood, previous studies suggest that changes in nutrient regime—particularly in phosphorus forms and their bioavailability—may provide an important context for community reorganization [7,8]. In eutrophic coastal waters subject to excessive nitrogen loading, elevated N:P ratios and reduced dissolved inorganic phosphorus (DIP) are frequently observed, increasing the likelihood of phosphorus stress [9,10]. In such environments, dissolved organic phosphorus (DOP) can act as an alternative phosphorus source for phytoplankton production, while variation in its composition and bioavailability may further modulate phosphorus-use efficiency among phytoplankton groups and potentially shape community structure [11,12]. In general, diatoms are more competitive under sufficient DIP supply, whereas dinoflagellates may be better adapted to low-DIP conditions and greater reliance on organic phosphorus sources [8,13]. For example, in DIP-limited systems such as the Changjiang Estuary, shifts from diatom to dinoflagellate dominance have been associated with the ability of some dinoflagellates to utilize DOP under inorganic phosphorus scarcity [7,14]. Meanwhile, nutrient stoichiometry and hydrodynamic conditions may further influence seasonal phytoplankton community patterns by altering resource supply, water-column stability, and the competitive balance among major groups [15,16].

This issue is further complicated when apparent dinoflagellate dominance is driven primarily by *Noctiluca scintillans*. Although taxonomically classified as a dinoflagellate, *N. scintillans* is predominantly heterotrophic and differs fundamentally from autotrophic dinoflagellates in both trophic strategy and ecological role [17]. Previous studies indicate that blooms of *N. scintillans* are not typically driven by a single nutrient factor, but instead reflect the combined effects of nutrient conditions, prey supply, and physical accumulation processes. For instance, in semi-enclosed bays of Hong Kong, the population dynamics of *N. scintillans* have been linked to the availability of phytoplankton prey, particularly diatoms, and are further modulated by wind, tides, and circulation [17]. Similarly, in Sagami Bay, Japan, blooms of *N. scintillans* have been associated with low rainfall and wind-driven convergence [18]. Accordingly, when apparent spring dinoflagellate dominance is driven largely by *N. scintillans*, it should not be interpreted simply as an increase in autotrophic dinoflagellate abundance, but rather as a distinct community state shaped by nutrient structure, the reduced competitive advantage of autotrophic taxa, food-web interactions, and physical forcing.

Liaodong Bay, a semi-enclosed embayment in the northern Bohai Sea, has long been affected by eutrophication associated with riverine inputs, industrial discharge, and aquaculture activities, resulting in elevated nutrient loads and frequent phytoplankton blooms [19]. Although previous studies in Liaodong Bay have described nutrient distributions, water quality, and local bloom events, systematic evidence on spring-to-summer phytoplankton community reorganization remains limited, and the environmental conditions associated with this reorganization remain poorly resolved. To address this gap, we conducted field surveys in spring and summer 2025 to characterize seasonal changes in phytoplankton community structure and associated environmental conditions. We aimed to (i) characterize spring-to-summer changes in phytoplankton community structure in Liaodong Bay and (ii) assess the environmental conditions associated with the observed community reorganization, with particular emphasis on phosphorus forms and nutrient stoichiometry.

## 2. Materials and Methods

### 2.1. Study Area

Liaodong Bay, one of the three major bays of the Bohai Sea, is a shallow, semi-enclosed marine system covering approximately 3.0 × 10^4^ km^2^, with a mean depth of approximately 16 m. The region is strongly influenced by the East Asian monsoon and exhibits pronounced seasonal variability, including rainy summers and cold winters. Several rivers discharge into the bay, among which the Liaohe and Daliao rivers represent important sources of terrestrial nutrients. The semi-enclosed topography and relatively weak hydrodynamic exchange may favor nutrient retention in coastal and inner-bay waters. Consequently, sustained riverine and anthropogenic nutrient inputs have contributed to persistent eutrophication and an elevated risk of harmful algal blooms [19].

### 2.2. Sample Collection

**Figure 1:**
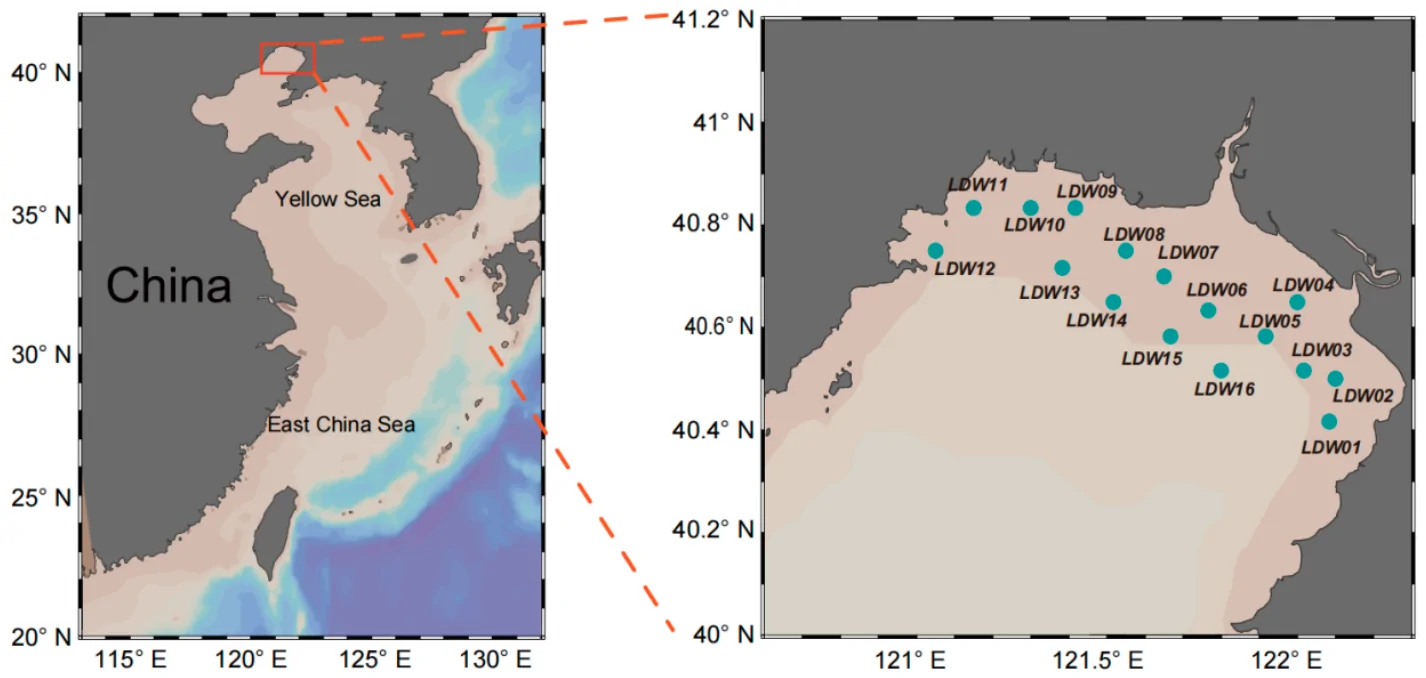
Study area and sampling stations in Liaodong Bay.

Two field surveys were conducted at 16 stations in Liaodong Bay in spring and summer 2025 (Figure 1). At each station, surface seawater was collected at a depth of approximately 0.5 m using Niskin bottles for phytoplankton and environmental analyses. For nutrient measurements, 500 mL of seawater was filtered through pre-cleaned 0.45 μm cellulose acetate membranes, and the filtrates were analyzed for total dissolved nitrogen (TDN), total dissolved phosphorus (TDP), ammonium nitrogen (NH₄-N), nitrate nitrogen (NO₃-N), nitrite nitrogen (NO₂-N), DIP, and dissolved silicate (DSi). Separate seawater samples were filtered for chlorophyll a (Chl a) determination. Nutrient filtrates and Chl a filters were immediately frozen on board and stored at −20 °C until analysis. For phytoplankton identification and enumeration, approximately 500 mL of seawater was fixed with Lugol’s iodine solution to a final concentration of 1–1.5% and stored in the dark. Sea surface temperature (SST), sea surface salinity (SSS), dissolved oxygen (DO), and pH were measured in situ using a multiparameter water-quality sonde (YSI 6600, YSI Incorporated, Yellow Springs, OH, USA). Chemical oxygen demand (COD) was determined using the permanganate index method, and suspended solids (SS) was measured gravimetrically after filtration and drying at 105 °C to constant weight.

### 2.3. Sample Analysis

Dissolved inorganic nutrients (NH_4_-N, NO_3_-N, NO_2_-N, DIP, and DSi) were measured using an automated nutrient analyzer (QuAAtro, SEAL Analytical, Norderstedt, Germany). Prior to analysis, TDN and TDP samples were digested with alkaline potassium persulfate (121 °C, 30 min) to convert dissolved organic nitrogen (DON) and DOP into inorganic forms. DIN was defined as the sum of NH_4_-N, NO_3_-N, and NO_2_-N. DON and DOP were calculated as the differences between TDN and DIN and between TDP and DIP, respectively. The method detection limits for DIP and TDP were 0.03 and 0.10 μmol P L^−1^, respectively. For a conservative assessment of DOP uncertainty, 0.03 μmol P L^−1^ was used as the analytical uncertainty estimate for both DIP and TDP, based on replicate measurements conducted using the same analytical procedures. The combined uncertainty was propagated using the root-sum-of-squares method. Chl a concentrations were determined by acetone extraction. Filters were extracted in 90% acetone at 4 °C in the dark for 24 h, followed by centrifugation. The absorbance of the supernatant was measured at multiple wavelengths using a UV–visible spectrophotometer, and Chl a concentrations were calculated [20]. For phytoplankton community analysis, Lugol-fixed samples were thoroughly mixed prior to examination. Phytoplankton were identified and enumerated under an inverted microscope following standard taxonomic procedures. At each station, the relative abundance of each species was expressed as a percentage of the total phytoplankton abundance. Seasonal mean relative abundance was calculated by averaging these station-specific percentages across all stations, with a value of 0% assigned at stations where the species was not detected. Species with a seasonal mean relative abundance >2% were considered dominant species. Additionally, although *N. scintillans* is predominantly heterotrophic, it is taxonomically classified as a dinoflagellate and is commonly enumerated in microscopy-based plankton community studies [21,22,23]. Therefore, it was retained in the community dataset and ordination analyses for consistency with conventional taxonomic treatment and previous studies. In the subsequent ecological interpretation, however, *N. scintillans* was treated as a distinct heterotrophic dinoflagellate and was distinguished from autotrophic dinoflagellates.

### 2.4. Data Analysis

All statistical analyses and figure preparation were conducted primarily in R version 4.5.1 [24]. Differences in environmental variables and key biological indicators between spring and summer at the same sampling stations were assessed using paired t-tests when the assumptions of normality were satisfied; otherwise, Wilcoxon signed-rank tests were used. Relationships between the abundances of diatoms and dinoflagellates and environmental variables were examined using Pearson or Spearman correlation analyses. Differences in phytoplankton community composition between spring and summer were visualized using non-metric multidimensional scaling (NMDS) based on a Bray–Curtis dissimilarity matrix. Differences in community composition were further tested using permutational multivariate analysis of variance (PERMANOVA), implemented in the vegan package version 2.7.2 [25]. To evaluate whether variation in within-group dispersion could influence the PERMANOVA results, the homogeneity of multivariate dispersions was tested using betadisper. Similarity percentage analysis (SIMPER) was then performed to identify the species contributing most to the compositional dissimilarity between spring and summer. Phytoplankton α-diversity was characterized using the Shannon diversity index and Pielou’s evenness index, and differences between spring and summer were compared. To examine relationships between phytoplankton community structure and environmental variables, detrended correspondence analysis (DCA) was first performed to estimate gradient lengths; the results indicated that redundancy analysis (RDA) was appropriate. Prior to ordination, species abundance data were Hellinger-transformed, and environmental variables were standardized [25]. Multicollinearity among the explanatory variables was assessed using variance inflation factors (VIFs), and variables with high VIF values were sequentially removed until all retained variables had VIF values below 10. Based on this procedure, pH, DO, NO_3_-N, and NO_2_-N were excluded from the final RDA model. The sampling station map was produced using Ocean Data View [26], and all other figures were prepared in R. Statistical significance was set at *p* < 0.05 for all analyses.

## 3. Results

### 3.1. Seasonal Variation in Environmental Variables

**Figure 2:**
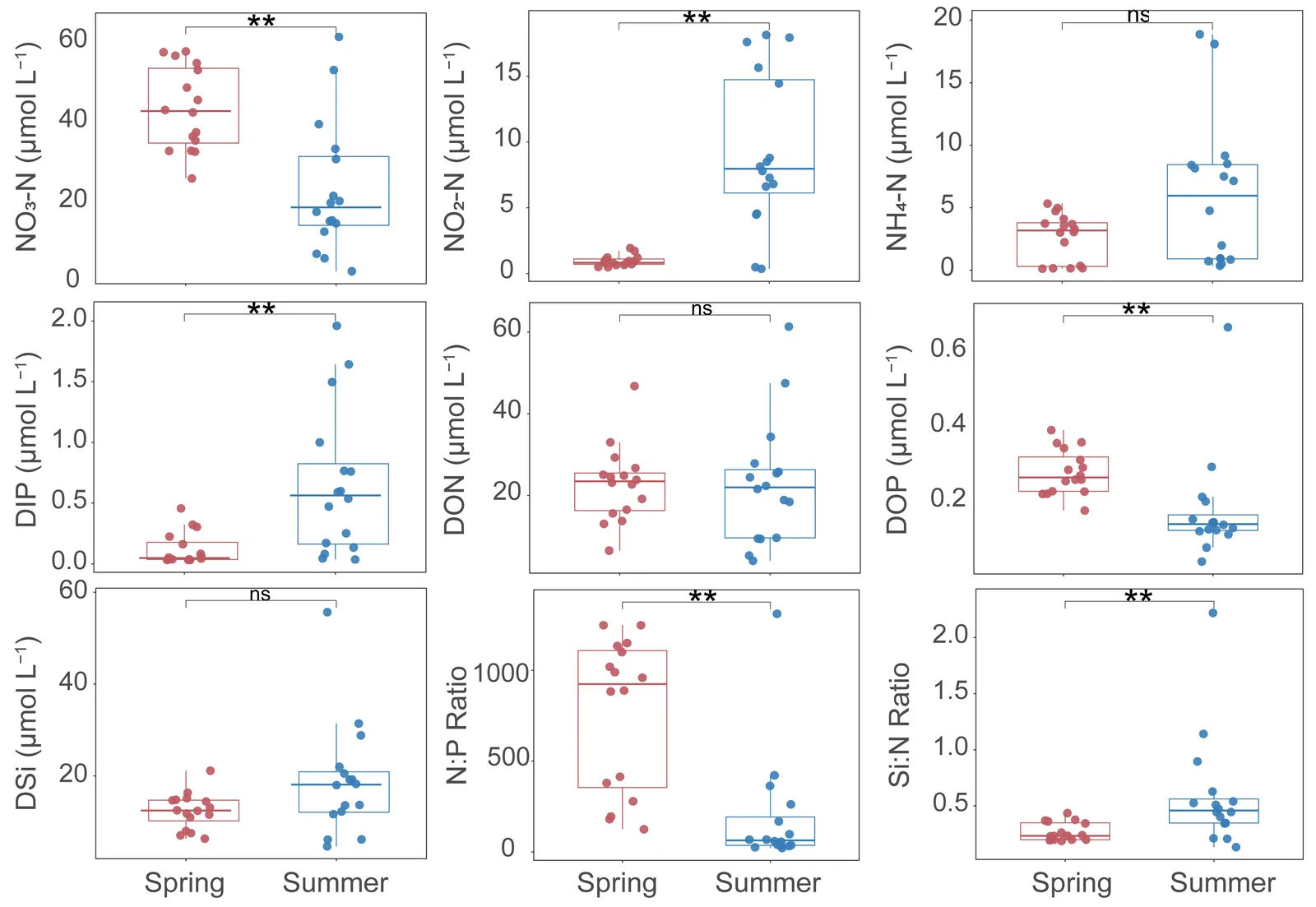
Seasonal variations in nutrient concentrations in surface waters of Liaodong Bay. *p* < 0.01 is denoted by **, and ns indicates no significant difference.

Surface-water nutrient composition and stoichiometric ratios in Liaodong Bay differed markedly between spring and summer (Figure 2). Among DIN forms, NO_3_-N was dominant in both seasons and occurred at a significantly higher concentration in spring than in summer (43.46 ± 10.08 vs. 23.33 ± 16.55 μmol L^−1^, *p* < 0.01). By contrast, NO_2_-N and NH_4_-N were present at much lower concentrations than NO_3_-N, but both were significantly higher in summer (*p* < 0.01). DIP increased significantly from 0.12 ± 0.12 μmol L^−1^ in spring to 0.66 ± 0.59 μmol L^−1^ in summer (*p* < 0.01), whereas DOP showed the opposite pattern, with significantly higher concentrations in spring than in summer (0.28 ± 0.06 vs. 0.18 ± 0.14 μmol L^−1^, *p* < 0.01). In spring, the mean DOP–DIP difference was 0.157 μmol L^−1^, exceeding the propagated uncertainty estimate of 0.067 μmol L^−1^ and supporting the robustness of the observed DOP dominance. DON showed no significant seasonal difference (*p* > 0.05). The N:P ratio was significantly higher in spring than in summer (*p* < 0.01). Although DSi showed no significant seasonal difference (*p* > 0.05), its concentration tended to be higher in summer, and the Si:N ratio was also significantly higher in summer (*p* < 0.01). Overall, the nutrient regime in the surface waters of Liaodong Bay shifted from relatively high NO_3_-N, DOP, and N:P ratios in spring to relatively high DIP, NO_2_-N, and Si:N ratios in summer.

**Figure 3:**
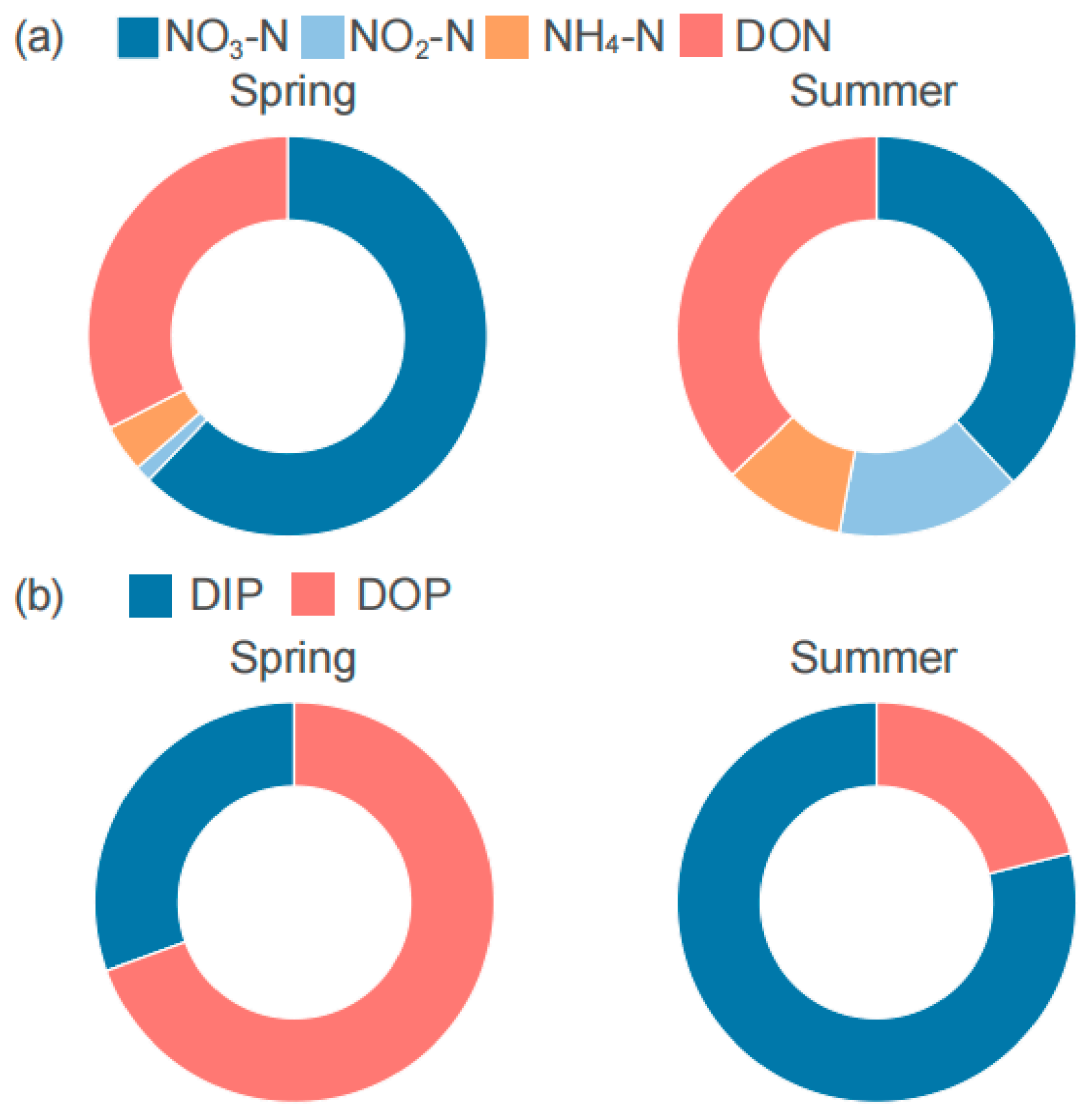
Seasonal variations in nitrogen (**a**) and phosphorus (**b**) forms in surface waters of Liaodong Bay.

The relative contributions of dissolved nitrogen and phosphorus forms differed markedly between spring and summer (Figure 3). In spring, total dissolved nitrogen was dominated by NO_3_-N (62.15%), followed by DON (32.50%), whereas NO_2_-N and NH_4_-N made only minor contributions (1.35% and 4.00%, respectively). In summer, the contribution of NO_3_-N declined to 35.17%, while DON accounted for 37.58%; at the same time, the contributions of NO_2_-N and NH_4_-N increased to 15.79% and 11.46%, respectively. For phosphorus, DOP was the dominant form in spring, accounting for 75.03%, whereas DIP accounted for only 24.97%. In summer, however, the contribution of DIP increased to 65.48%, while that of DOP declined to 34.52%. Overall, the dissolved phosphorus pool shifted from DOP dominance in spring to DIP dominance in summer.

**Figure 4:**
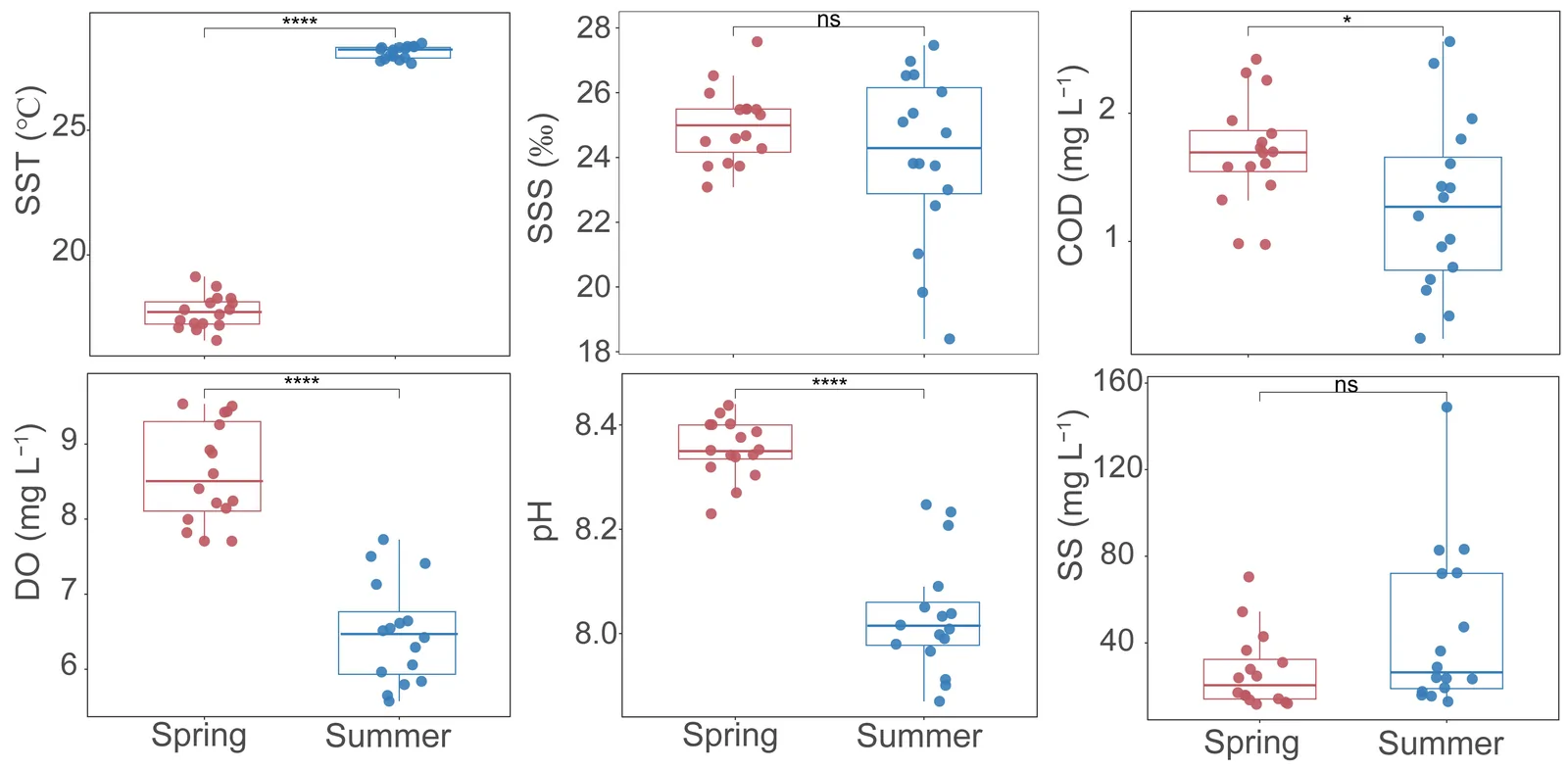
Seasonal variations in surface temperature, salinity, dissolved oxygen, pH, COD, and suspended solids in Liaodong Bay during spring and summer. *p* < 0.05 is denoted by *, *p* < 0.0001 by ****, and ns indicates no significant difference.

Surface physicochemical variables in Liaodong Bay also differed markedly between spring and summer (Figure 4). SST was significantly higher in summer than in spring, increasing from 17.72 °C to 28.12 °C (*p* < 0.01). In contrast, DO declined significantly to 6.48 mg L^−1^ in summer, which was markedly lower than the spring level (*p* < 0.01). pH also showed significant seasonal variation, with lower values in summer than in spring (8.03 vs. 8.35, *p* < 0.01). In comparison, SSS did not differ significantly between the two seasons (*p* > 0.05). In addition, COD was significantly higher in spring than in summer (*p* < 0.05), whereas SS was generally higher in summer, although the seasonal difference was not statistically significant (*p* > 0.05).

### 3.2. Seasonal Variation in Total Phytoplankton Abundance and Major Taxonomic Groups

**Figure 5:**
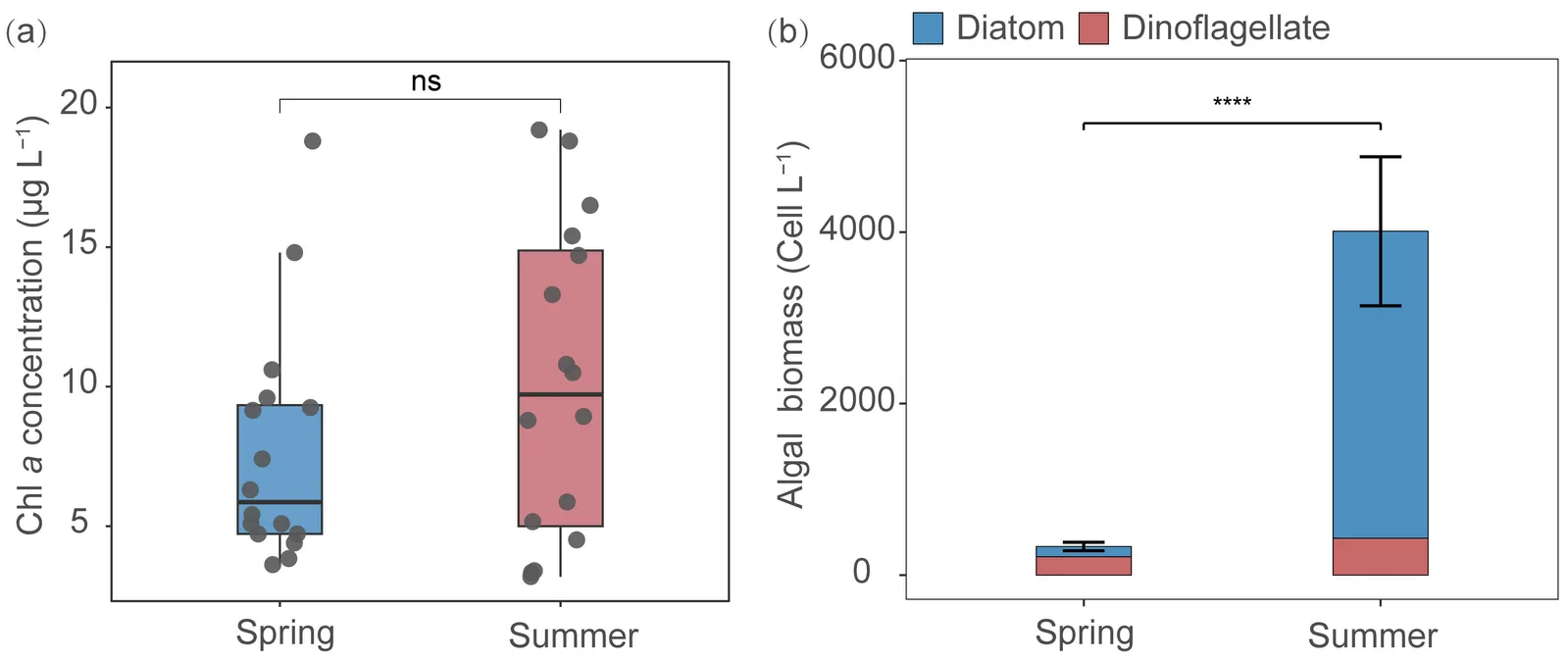
Seasonal variations in chlorophyll a (**a**) and phytoplankton abundance (**b**) in Liaodong Bay. *p* < 0.0001 is denoted by ****, and ns indicates no significant difference.

Seasonal variations in Chl a concentration and phytoplankton abundance between spring and summer are shown in Figure 5. Mean Chl a concentration was higher in summer than in spring, but the difference was not statistically significant (7.16 μg L^−1^ in spring vs. 10.15 μg L^−1^ in summer, *p* > 0.05). In contrast, total phytoplankton abundance differed significantly between seasons, with a mean cell density of 4010.82 cells L^−1^ in summer, approximately 12 times that in spring (333.98 cells L^−1^). Analysis of major taxonomic groups showed that dinoflagellates were more abundant than diatoms in spring (213.2 vs. 120.7 cells L^−1^), whereas this pattern reversed in summer, with diatoms exceeding dinoflagellates (3581.1 vs. 429.7 cells L^−1^; *p* < 0.05). Thus, summer diatom dominance primarily reflected a disproportionate increase in diatom abundance rather than a decline in dinoflagellate abundance.

### 3.3. Changes in Phytoplankton Community Composition and Dominant Species

**Figure 6:**
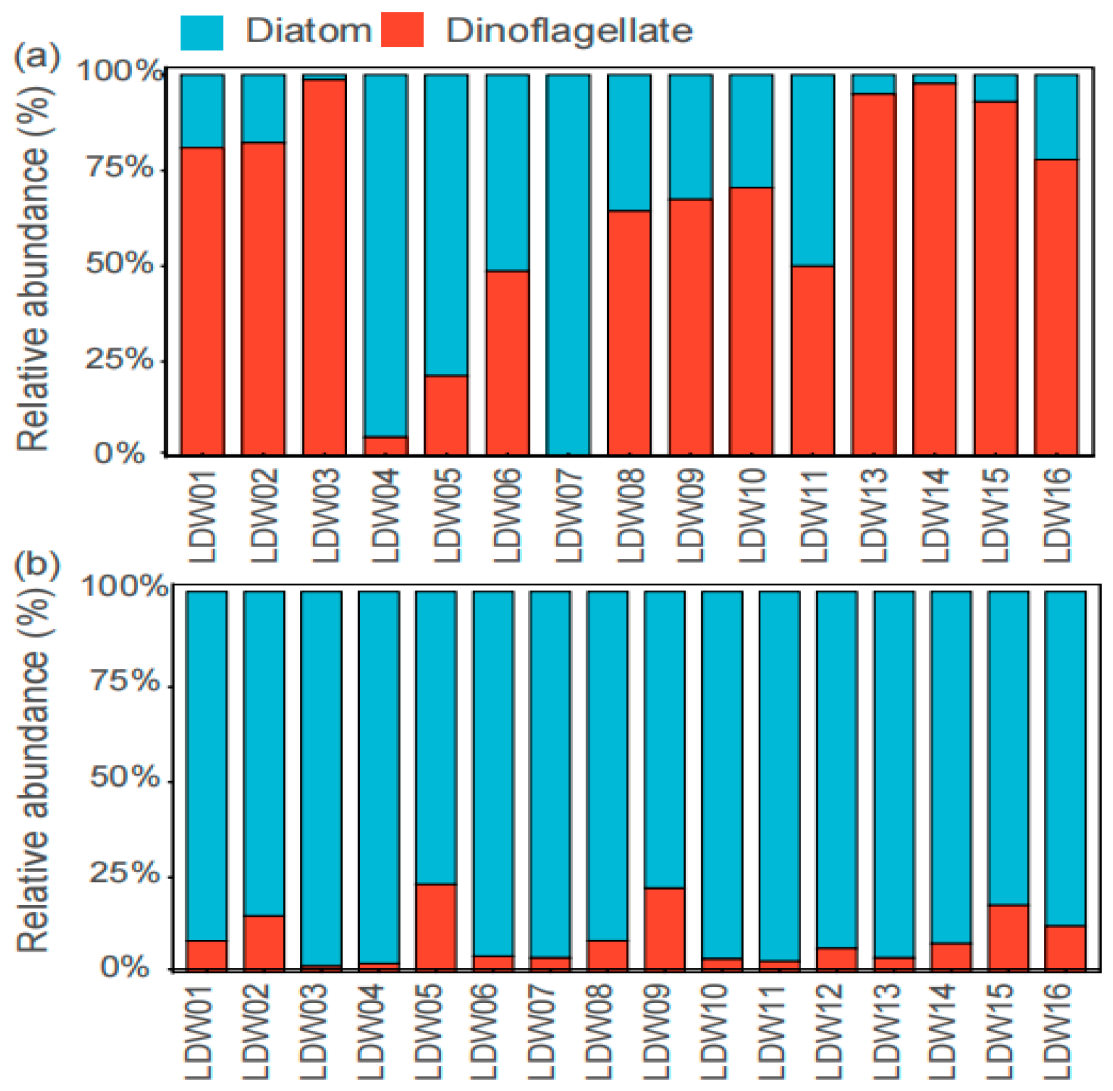
Seasonal variations in the relative abundance of major phytoplankton groups (diatoms and dinoflagellates) in Liaodong Bay. (**a**) Spring; (**b**) Summer.

**Figure 7:**
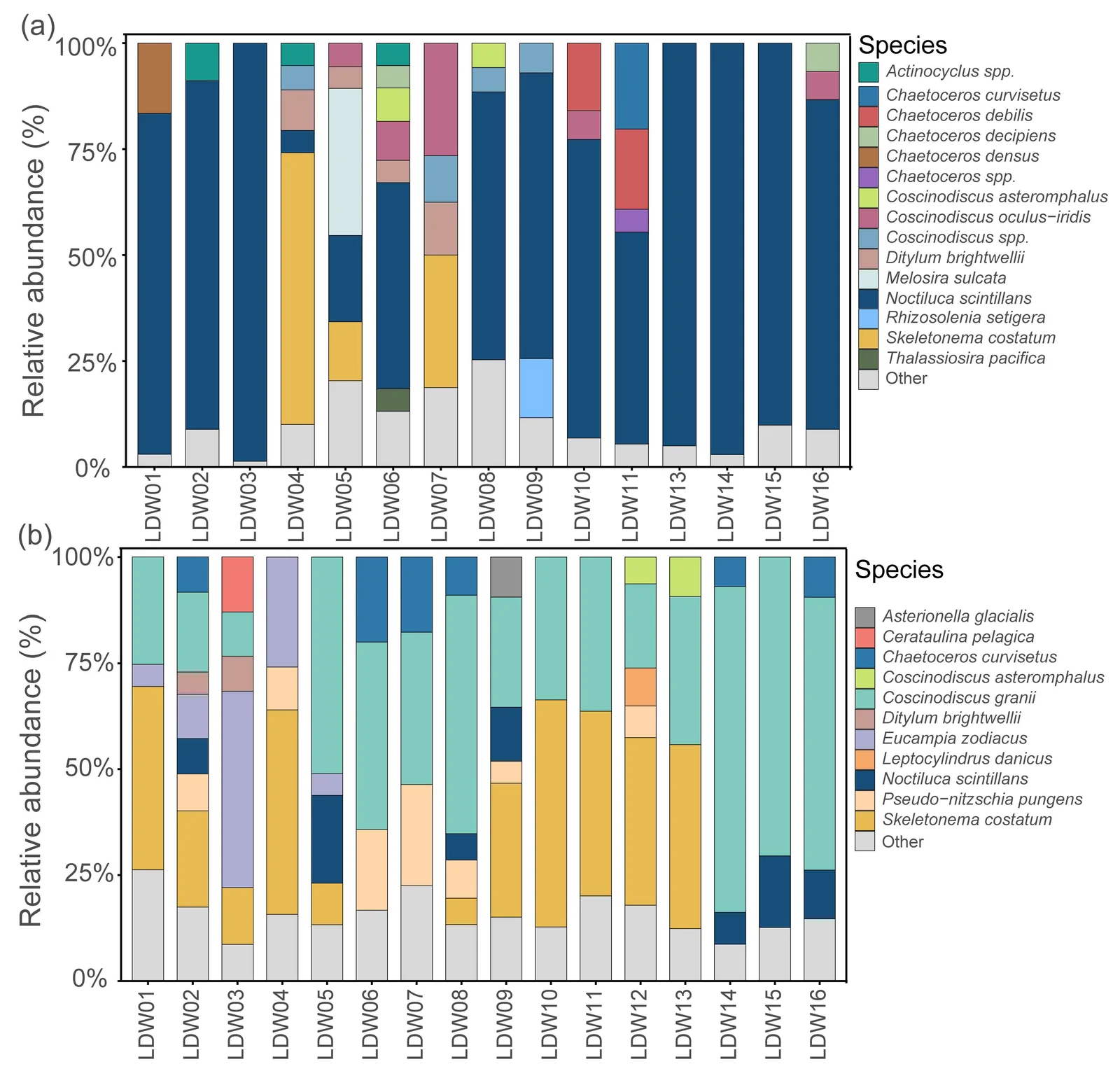
Seasonal variations in phytoplankton community structure in Liaodong Bay. (**a**) Spring; (**b**) Summer.

In spring, the community showed apparent dinoflagellate dominance, with dinoflagellates accounting for 63.51% of total abundance and diatoms accounting for 36.49% (Figure 6 and Figure 7). In summer, however, the community shifted to strong diatom dominance, with the relative abundance of diatoms increasing to 91.02% and that of dinoflagellates declining to 8.98%. In terms of species composition, the apparent spring dinoflagellate dominance was almost entirely attributable to the heterotrophic dinoflagellate *N. scintillans*, which accounted for 63.11% of total abundance and dominated at most stations. Other species made comparatively minor contributions, with only *Skeletonema costatum* (7.28%) and *Coscinodiscus oculus-iridis* (4.16%) contributing appreciably. In contrast, multiple diatom species co-dominated the summer community. Among them, *Coscinodiscus granii* and *S. costatum* accounted for 37.93% and 22.62% of total abundance, respectively, whereas the proportion of *N. scintillans* declined to 6.29%.

### 3.4. Phytoplankton Community Diversity, Overall Differentiation, and Key Contributing Species

**Figure 8:**
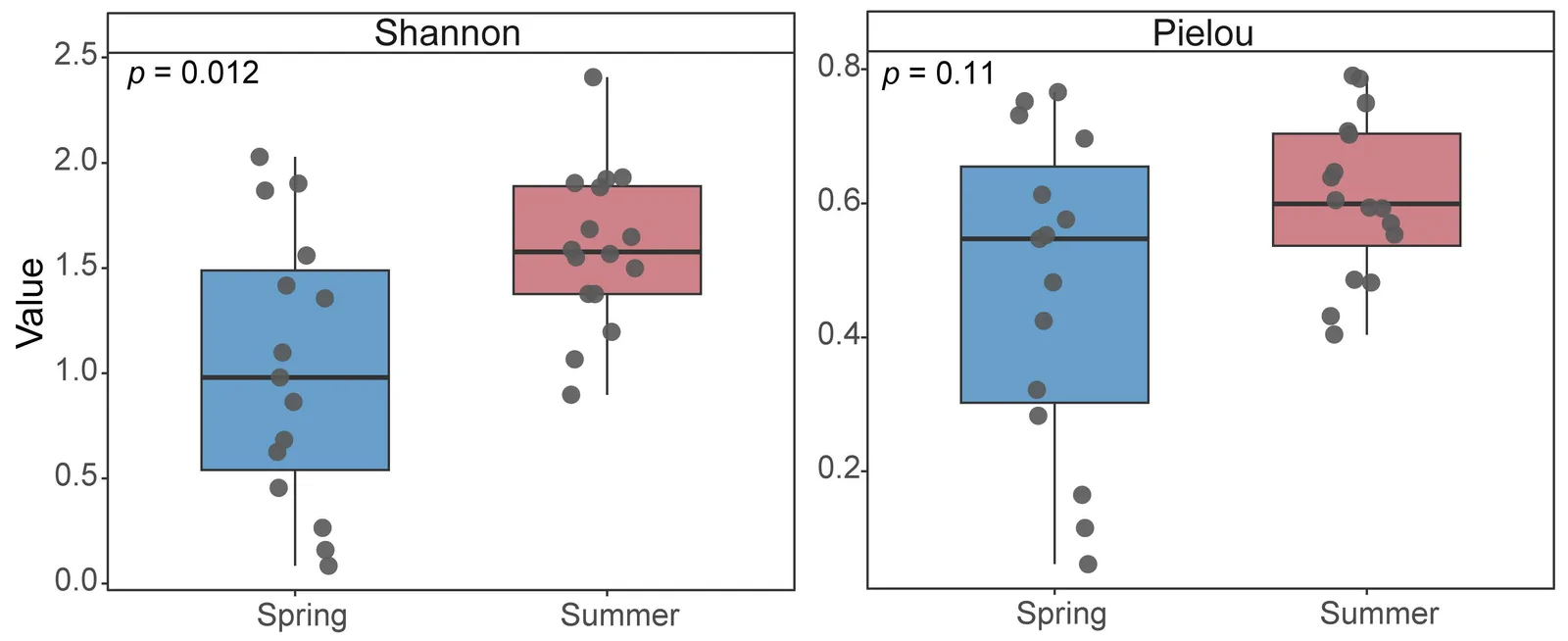
Seasonal variations in phytoplankton alpha diversity in Liaodong Bay.

**Figure 9:**
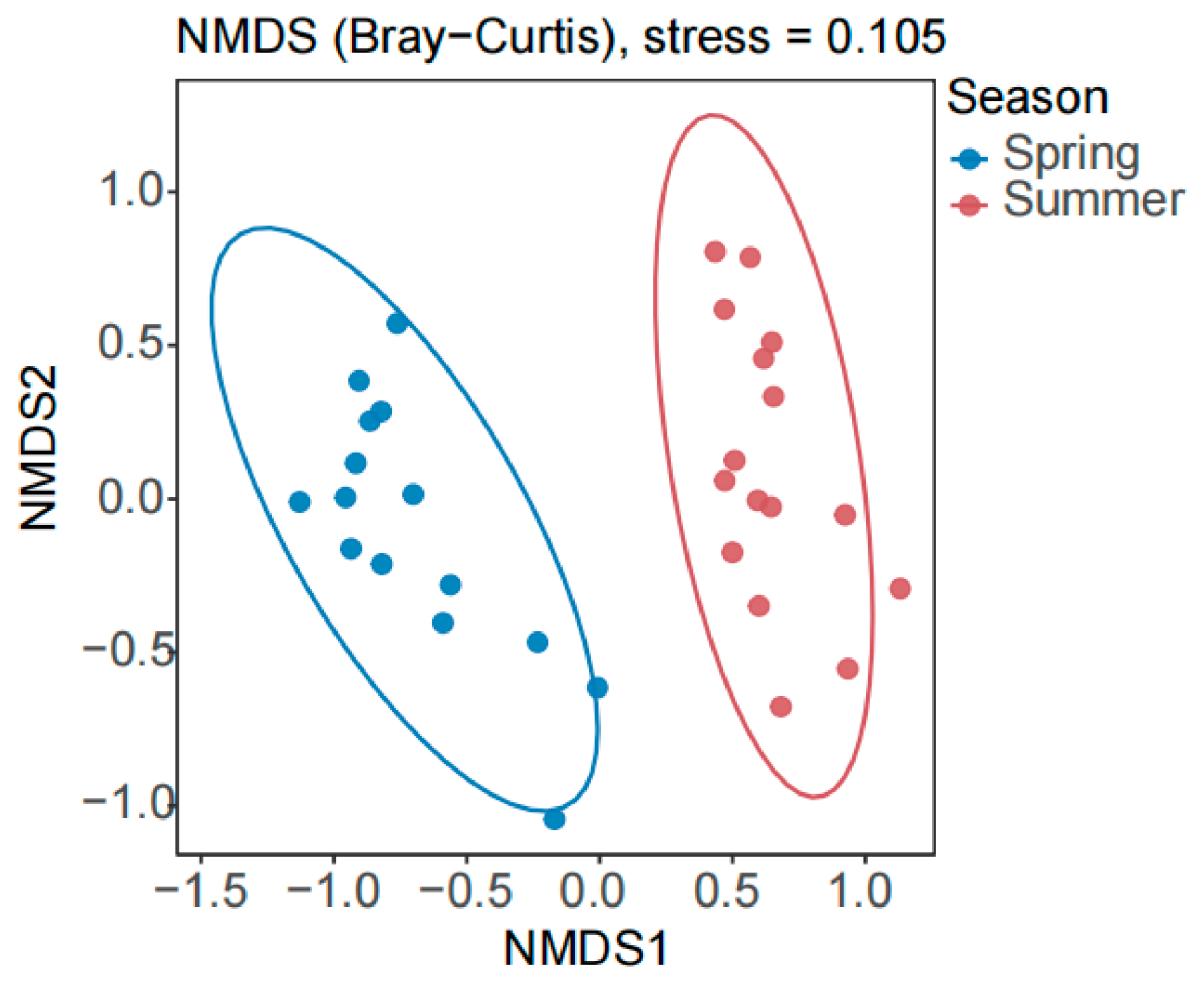
NMDS ordination of phytoplankton communities in spring and summer in Liaodong Bay.

The α-diversity of the phytoplankton community showed seasonal differences between spring and summer (Figure 8). The Shannon index was significantly higher in summer than in spring (*p* < 0.05), likely reflecting reduced dominance by a single species and greater community evenness in summer. The Pielou evenness index also increased in summer, although the seasonal difference was not statistically significant (*p* > 0.05). NMDS ordination further showed a clear separation between spring and summer samples in two-dimensional ordination space (Figure 9), indicating distinct seasonal differentiation in phytoplankton community composition. Additionally, PERMANOVA confirmed a significant difference in community structure between spring and summer (R^2^ = 0.395, *p* = 0.001), with season explaining 39.5% of the variation in community composition. The homogeneity test for multivariate dispersions (betadisper; *p* = 0.964) indicated that the observed seasonal differentiation was driven primarily by differences in community composition between groups, rather than by differences in within-group dispersion.

To identify the species contributing most to the seasonal differentiation in community composition between spring and summer, similarity percentage analysis (SIMPER) was performed on the community data (Table 1). The top 10 contributing species collectively explained 69.10% of the dissimilarity between the spring and summer communities. Among them, *C. granii* had the highest average contribution (0.178). It was not detected in spring but reached a mean abundance of 2055.34 cells L^−1^ in summer, making it the primary contributor to seasonal community differentiation. *S. costatum* and *Pseudo-nitzschia pungens* ranked next, with average contributions of 0.101 and 0.064, respectively. Both species were substantially more abundant in summer than in spring, increasing from 28.25 and 0 cells L^−1^ to 478.46 and 279.30 cells L^−1^, respectively. In addition, *Chaetoceros curvisetus* and *Eucampia zodiacus* also contributed substantially to the seasonal difference, with mean summer abundances of 277.10 and 123.10 cells L^−1^, respectively. Notably, *N. scintillans* was also among the major contributing species, with a contribution value of 0.062.

### 3.5. Relationships Between Community Structure and Environmental Factors

**Figure 10:**
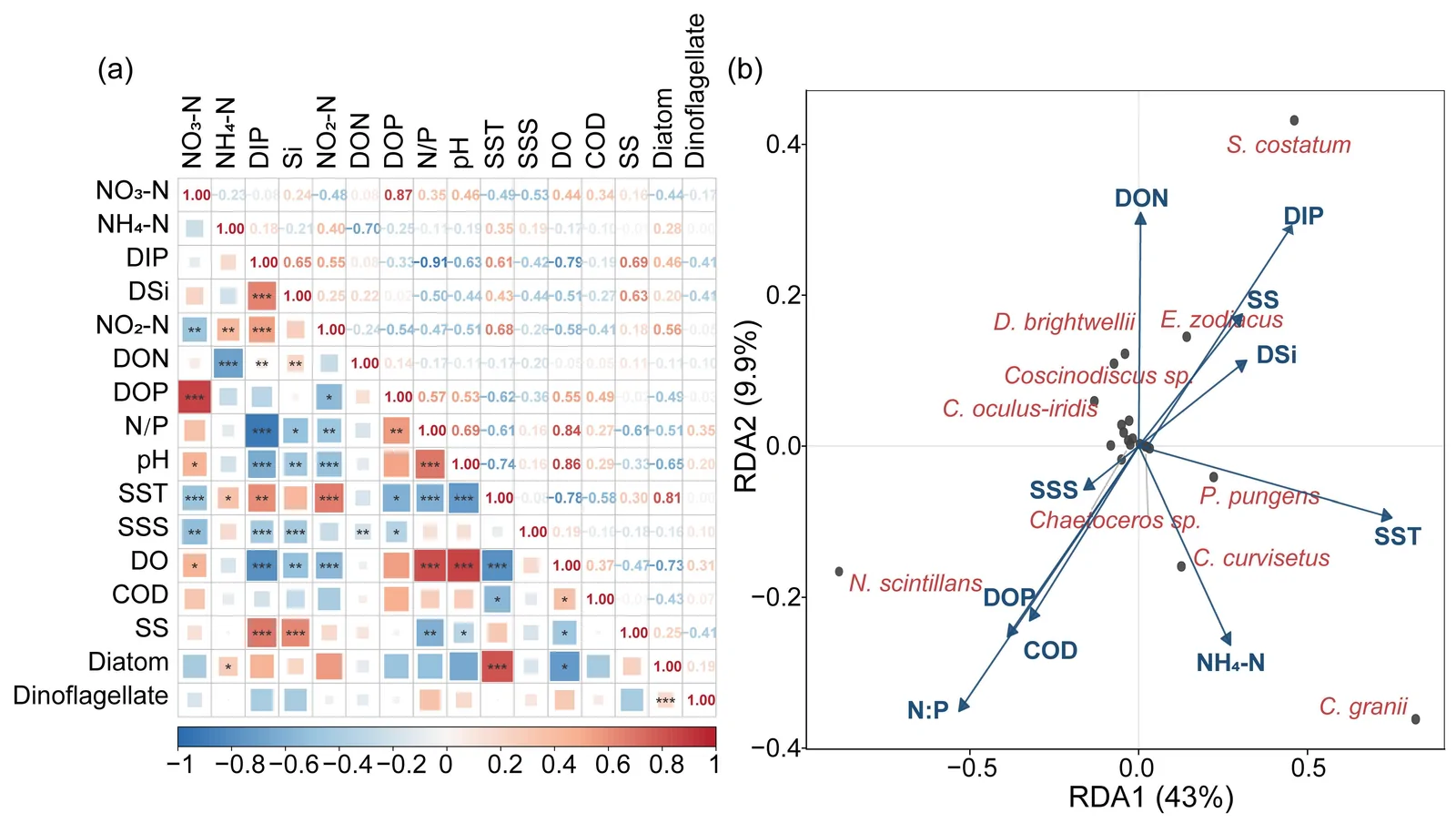
Relationships between phytoplankton groups, dominant species, and key environmental factors. (**a**) Correlation analysis; (**b**) redundancy analysis. *p* < 0.05 is denoted by *, *p* < 0.01 by **, *p* < 0.001 by ***, and ns indicates no significant difference.

The relationships between community structure and environmental factors are shown in Figure 10. Correlation analysis showed that diatom abundance was significantly positively correlated with NH_4_-N and SST (*p* < 0.05), and significantly negatively correlated with DO (*p* < 0.01). Diatom abundance also tended to be positively correlated with DIP and DSi and negatively correlated with DOP and N:P, but these relationships were not statistically significant (*p* > 0.05). In contrast, dinoflagellate abundance tended to be positively correlated with DOP and N:P and negatively correlated with DIP, DSi, and SS; however, these relationships were also not statistically significant (*p* > 0.05).

Redundancy analysis (RDA) further described broad multivariate relationships between dominant species and environmental gradients (Figure 10b). The spring-dominant species *N. scintillans* was positioned toward the DOP, COD, and N:P vectors and was relatively separated from the gradients represented by DIP, DSi, and NH_4_-N, suggesting broad alignment with relatively higher DOP and N:P conditions. The dominant diatom species in summer showed different distribution patterns. *S. costatum* and *E. zodiacus* were positioned closer to the DIP and DSi gradients, whereas *Ditylum brightwellii* was more closely aligned with the DON gradient. *C. granii* and *C. curvisetus* were distributed toward the NH_4_-N and SST gradients, while *P. pungens* was positioned mainly toward the SST gradient.

## 4. Discussion

### 4.1. Pronounced Spring-to-Summer Reorganization of the Phytoplankton Community in Liaodong Bay

This study showed that the phytoplankton community in Liaodong Bay underwent marked spring-to-summer reorganization, shifting from apparent dinoflagellate dominance driven by the heterotrophic dinoflagellate *N. scintillans* in spring to a diatom-dominated assemblage in summer (Figure 5 and Figure 6). This pattern contrasts with the seasonal succession commonly reported in temperate coastal waters, where diatoms typically dominate in spring and dinoflagellates become more prominent in summer [27], indicating that the phytoplankton community in Liaodong Bay exhibited a distinct spring-to-summer pattern during the study period. Beyond this shift in dominant groups, the Shannon index was significantly higher in summer than in spring (*p* < 0.05), while multivariate analyses confirmed clear seasonal differentiation in community composition (Figure 8 and Figure 9). SIMPER further indicated that this differentiation was largely associated with the summer expansion of several dominant diatom taxa (Table 1). Collectively, these results indicate that the observed seasonal change involved not merely fluctuations in abundance, but a broader reorganization of dominant groups, diversity, and dominant-species composition.

Ecologically, the apparent spring dinoflagellate dominance represented a heterotrophic *N. scintillans*-dominated state rather than a conventional assemblage composed of multiple autotrophic dinoflagellates (Figure 7). In spring, *N. scintillans* accounted for 99.56% of dinoflagellate abundance and 63.11% of total phytoplankton abundance, indicating pronounced single-species dominance, consistent with the lower Shannon index observed in spring. In contrast, summer dominance was shared among several diatom taxa, including *C. granii*, *S. costatum*, and *P. pungens*, indicating a transition from strong single-species dominance to multi-diatom co-dominance.

### 4.2. Nutrient-Regime Shifts Associated with Spring-to-Summer Phytoplankton Reorganization

**Figure 11:**
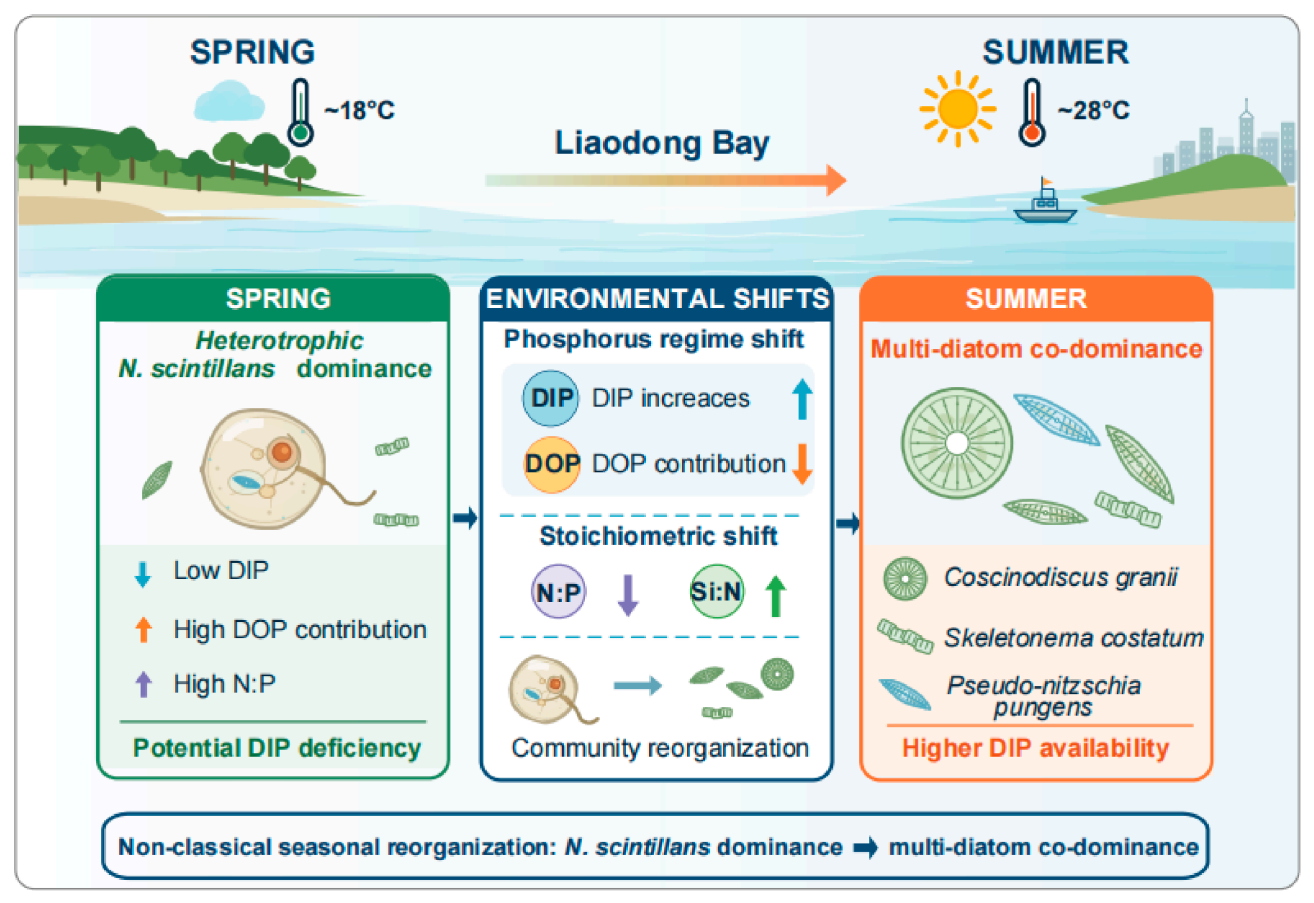
Conceptual summary of the non-classical spring-to-summer phytoplankton community reorganization in Liaodong Bay and associated shifts in phosphorus regime and nutrient stoichiometry.

Marked shifts in nutrient conditions accompanied the spring-to-summer reorganization of the phytoplankton community, with phosphorus forms and nutrient stoichiometry providing an important environmental context for this reorganization (Figure 11). In surface waters, DIP concentration increased from 0.12 μmol L^−1^ in spring to 0.66 μmol L^−1^ in summer, accompanied by a shift in the dominant phosphorus form from DOP in spring (75.03%) to DIP in summer (65.48%) (Figure 2 and Figure 3). In addition, the N:P ratio was significantly higher in spring than in summer, whereas the Si:N ratio showed the opposite pattern (*p* < 0.01). Taken together, these changes indicate a shift from a spring regime characterized by low DIP, a high relative contribution of DOP, and high N:P to a summer regime characterized by higher DIP, lower N:P, and higher Si:N. Importantly, DIP is generally considered readily available to most phytoplankton taxa, whereas DOP bioavailability varies with its molecular composition and taxon-specific capacities for hydrolysis and uptake [7,8]. Thus, spring DOP dominance did not necessarily provide sufficient bioavailable P for diatoms, whereas summer DIP dominance indicated greater inorganic P availability and conditions more favorable for diatom expansion. These contrasting nutrient contexts were broadly reflected in the statistical analyses, although most nutrient-community correlations were not statistically significant. Diatom abundance showed positive trends with DIP, DSi, Si:N, and NH_4_-N and negative trends with DOP and N:P, whereas the spring *N. scintillans*-dominated assemblage coincided with a higher DOP contribution and a higher N:P ratio. RDA showed a similar multivariate pattern, with *N. scintillans* positioned toward the DOP and N:P gradients, whereas dominant summer diatoms were more closely aligned with DIP, DSi, and NH_4_-N gradients (Figure 10).

Spring dominance of *N. scintillans* may have been associated with the combined effects of the prevailing nutrient regime and other environmental conditions. In spring, low DIP coupled with a high N:P ratio suggested potential relative inorganic phosphorus deficiency and a nutrient regime less conducive to diatom dominance (Figure 2) [16]. By contrast, as a heterotrophic dinoflagellate, *N. scintillans* is less directly dependent on inorganic nutrient uptake than autotrophic phytoplankton, and its high abundance may have been influenced by food availability and physical environmental conditions. Beyond nutrient conditions, temperature was also a relevant physical factor, as the spring SST of 17.72 °C was within the reported tolerable range for *N. scintillans* [17]. However, the emergence of multi-diatom co-dominance under warmer summer conditions contrasted with the commonly observed spring diatom–summer dinoflagellate pattern in temperate coastal waters, suggesting that temperature alone could not fully explain the observed seasonal reorganization [4]. Additionally, biological interactions and hydrodynamic conditions may also have contributed, as previous studies have shown that *N. scintillans* consumes diverse planktonic organisms and organic detritus, and that its blooms are often associated with low wind speed and reduced hydrodynamic disturbance [18,22]. Consistent with the potential role of prey availability, diatom taxa such as *S. costatum* and *C. oculus-iridis* remained present in spring and may have formed part of the prey field for *N. scintillans* (Figure 7), while their relatively low observed abundance may partly reflect grazing pressure from *N. scintillans*. Together, these observations suggest that the low-DIP, high-N:P nutrient regime may have been less favorable for diatom dominance, whereas prey availability, spring temperature, and hydrodynamic conditions may have contributed to the relative dominance of *N. scintillans*.

In contrast to spring dominance by *N. scintillans*, summer multi-diatom co-dominance coincided with higher DIP, lower N:P, higher Si:N, and a tendency toward higher DSi, a combination broadly consistent with a nutrient regime more favorable for diatom expansion (Figure 2 and Figure 7). This agrees with evidence that nutrient conditions can contribute substantially to diatom—dinoflagellate community patterns in temperate coastal waters [28]. Notably, summer seawater was characterized by higher temperature, lower DO, and a non-significant tendency toward higher SS (Figure 4). Previous studies indicate that organic matter remineralization, regeneration of bioavailable phosphorus under low-oxygen conditions, and particulate resuspension can each contribute to sustaining elevated inorganic phosphorus levels [29,30,31]. Although these processes were not directly measured in the present study, they may have contributed to higher summer DIP availability, thereby providing a resource background consistent with diatom expansion.

### 4.3. Ecological Implications of the Spring-to-Summer Community Shift

The spring-to-summer reorganization from a low-diversity spring community dominated by the heterotrophic dinoflagellate *N. scintillans* to a summer community co-dominated by multiple diatom species has important ecological implications. First, it suggests that spring-to-summer phytoplankton community reorganization in eutrophic semi-enclosed bays does not necessarily follow the commonly observed seasonal pattern in temperate coastal waters, and may also be associated with variations in nutrient forms and potential phosphorus bioavailability [16,32]. Second, the spring stage dominated by *N. scintillans* may represent a community state distinct from typical autotroph-dominated conditions. Given its heterotrophic feeding strategy, the high abundance of *N. scintillans* suggests that the spring community may have differed not only compositionally but also in food-web pathways and material cycling [22,33].

These findings are also relevant to ecological monitoring and management in eutrophic coastal waters. Traditional assessments of eutrophication have often focused on indicators such as DIN, DIP, or Chl a. However, previous studies have shown that total nutrient levels or single concentration-based indicators alone are often insufficient to fully explain phytoplankton community structure and its seasonal dynamics [34]. Consistent with this view, the spring-to-summer community reorganization coincided with changes in DIP, DOP contribution, N:P, and Si:N, together with a tendency toward higher DSi. Therefore, ecological monitoring in Liaodong Bay may benefit from considering phosphorus forms, silicate availability, nutrient stoichiometry, and the relative abundance of major functional groups, in addition to total nutrient concentrations and Chl a.

## 5. Conclusions

Based on two field surveys conducted in spring and summer 2025, this study shows that the phytoplankton community in Liaodong Bay underwent pronounced seasonal reorganization, shifting from a spring assemblage dominated by the heterotrophic dinoflagellate *N. scintillans* to a summer community co-dominated by multiple diatom species. This pattern differs from the classical seasonal pattern commonly reported in temperate coastal waters. Concurrently, the nutrient regime shifted from a spring pattern of low DIP, high DOP contribution, and high N:P, indicating potential relative inorganic phosphorus deficiency, to a summer pattern with higher DIP, lower N:P, a tendency toward higher DSi, and higher Si:N. Integrated analyses further suggest that the low-DIP, high-N:P nutrient background in spring may have been less favorable for diatom dominance, whereas the heterotrophic dinoflagellate *N. scintillans* may have been less directly constrained by inorganic nutrient availability, potentially contributing to its relative dominance in spring. By contrast, higher DIP and a tendency toward higher DSi in summer were consistent with a resource context favorable for the expansion of multiple dominant diatom taxa. Overall, these findings suggest that phosphorus regime and nutrient stoichiometry were broadly associated with the non-classical spring-to-summer phytoplankton community reorganization from *N. scintillans* dominance to multi-diatom co-dominance in Liaodong Bay.

## Figures and Tables

**Table 1:** Top 10 species contributing to the dissimilarity between spring and summer phytoplankton communities.

Species	Mean Contribution	Mean Abundance (Spring, Cells L^−1^)	Mean Abundance (Summer, Cells L^−1^)	Cumulative Contribution (%)	*p* Value
*C. granii*	0.1777	0.00	2055.34	21.23	0.001
*S. costatum*	0.1013	28.25	478.46	33.34	0.001
*P. pungens*	0.0641	0.00	279.30	41.01	0.001
*N. scintillans*	0.0615	212.01	368.67	48.36	0.999
*C. curvisetus*	0.0486	3.44	277.10	54.16	0.001
*E. zodiacus*	0.0374	0.00	123.10	58.64	0.001
*Protoperidinium* sp.	0.0283	0.00	26.85	62.03	0.001
*D. brightwellii*	0.0211	9.48	32.70	64.54	0.999
*Coscinodiscus* sp.	0.0203	7.88	35.73	66.97	0.380
*C. asteromphalus*	0.0178	6.65	17.83	69.10	0.952

## Data Availability

The original contributions presented in the study are included in the article. Further inquiries can be directed to the corresponding author.

## Abbreviations

The following abbreviations are used in this manuscript:

Chl a	Chlorophyll a
COD	Chemical oxygen demand
DCA	Detrended correspondence analysis
DIN	Dissolved inorganic nitrogen
DIP	Dissolved inorganic phosphorus
DON	Dissolved organic nitrogen
DOP	Dissolved organic phosphorus
DO	Dissolved oxygen
DSi	Dissolved silicate
NMDS	Non-metric multidimensional scaling
PERMANOVA	Permutational multivariate analysis of variance
RDA	Redundancy analysis
SIMPER	Similarity percentage analysis
SS	Suspended solids
SSS	Sea surface salinity
SST	Sea surface temperature
TDN	Total dissolved nitrogen
TDP	Total dissolved phosphorus
N:P	Nitrogen-to-phosphorus ratio
Si:N	Silicon-to-nitrogen ratio
Si:P	Silicon-to-phosphorus ratio
